# Intramedullary nailing versus plating for distal tibia fractures without articular involvement: a meta-analysis

**DOI:** 10.1186/s13018-015-0217-5

**Published:** 2015-06-16

**Authors:** Zhi Mao, Guoqi Wang, Lihai Zhang, Licheng Zhang, Shuo Chen, Hailong Du, Yanpeng Zhao, Peifu Tang

**Affiliations:** Department of Orthopedics, Chinese PLA General Hospital, No. 28 Fuxing Road, Beijing, 100853 People’s Republic of China; Department of Microsurgery, PLA 205 Hospital, Guta Area, JinZhou, Liaoning 121001 People’s Republic of China; Department of Medical Information, Chinese PLA General Hospital, Beijing, 100853 People’s Republic of China

**Keywords:** Tibia fracture, Distal tibia, Intramedullary nailing, Plating, Meta-analysis

## Abstract

**Background:**

The choice between intramedullary (IM) nailing or plating of distal tibia fractures without articular involvement remains controversial. A meta-analysis of randomized controlled trials (RCTs) and observational studies was performed to compare IM nailing with plating for distal tibia fractures without articular involvement and to determine the dominant strategy.

**Materials and methods:**

The PubMed, Embase, Cochrane Library databases, Chinese Wan-Fang Database, and China National Knowledge Infrastructure were searched.

**Results:**

Twenty-eight studies, which included 1863 fractures, met the eligible criteria. The meta-analysis did not identify a statistically significant difference between the two treatments in terms of the rate of deep infection, delayed union, removal of instrumentation, or secondary procedures either in the RCT or retrospective subgroups. IM nailing was associated with significantly more malunion events and a higher incidence of knee pain in the retrospective subgroup and across all the studies, but not significantly in the RCT subgroup, and a lower rate of delayed wound healing and superficial infection both in the RCT and retrospective subgroups relative to plating. A meta-analysis of the functional scores or questionnaires was not possible because of the considerable variation among the included studies, and no significant differences were observed.

**Conclusions:**

Evidence suggests that both IM nailing and plating are appropriate treatments as IM nailing shows lower rate of delayed wound healing and superficial infection and plating may avoid malunion and knee pain. These findings should be interpreted with caution, however, because of the heterogeneity of the study designs. Large, rigorous RCTs are required.

## Introduction

Distal tibia fractures without articular involvement are a common consequence of road traffic accidents or other high-energy injuries. These fractures differ from pilon fractures in terms of the mechanism of injury, management, and prognosis of the displaced bones [1]. The proximity of these fractures to the ankle joint leads to more complications than are seen with diaphyseal or middle-third injuries [2]. Thus, the treatment of distal tibia fractures remains problematic [3].

Intramedullary (IM) nailing and plating are the two major options for the treatment of distal tibia fractures. Indications of IM nailing are fractures in elderly people with thin skin or compromised soft tissue, patients with high risk of non-healing wound, and fractures with distal bone mass allowing insertion of two screws [4]. Plating is indicated for fractures with risk of malalignment, fractures with simple articular involvement, and fractures in which IM nailing is not amenable [4]. The two approaches have some theoretical disadvantages [3, 5]. IM nailing frequently results in malalignment, malunion, and knee pain [1, 6–9]. Tibia plating can achieve anatomic reduction, but it is associated with the risk of wound dehiscence and infection because of the minimal soft tissue cover over the anteromedial tibia [1, 10]. There have been some controlled clinical trials that directly compared the two methods [9, 11–32]. These trials also failed to show consistent results.

Several previous randomized controlled trials (RCTs) [2, 10, 33–35] have reported the outcomes of nailing versus plating treatment modalities. The limitations of observational studies were overcome in these RCTs by decreasing the bias through randomization. However, all of the RCTs had low numbers of patients. In 2013, Xue et al. [36] performed a meta-analysis and systematic review comparing nailing versus plating for the treatment of distal tibial metaphyseal fracture. Higher functional score and lower risk of infection were found in the nailing group. However, different categories of functional score were compounded, and no subgroup analysis was made as both RCT and retrospective studies were included. In 2014, a meta-analysis made by Kwok et al. [37] indicated that there was no significant difference between the use of a plate and nail regarding superficial infection and deep infection, but only four RCTs and four retrospective studies were included. Recently, some additional studies were reported [15, 18–31, 38], which will make the evidence more precise and reliable. As no consensus has been reached regarding the management of these fractures, the optimal treatment option for extra-articular distal tibia fractures remains controversial.

Therefore, we conducted this updated meta-analysis based on all relevant studies comparing IM nailing and plating in the treatment of distal tibia fractures without articular involvement. The aim of this meta-analysis was to assess the rate of complications and functional outcomes of the two methods.

## Materials and methods

### Inclusion and exclusion criteria

Studies were considered acceptable for inclusion in the meta-analysis if they met the following criteria: (1) patients more than 18 years of age who had undergone surgery for the following extra-articular distal tibia fractures: Association for the Study of Internal Fixation/American Orthopaedic Trauma Association (AO/OTA) type 42A to 42C, 43A, 43B1, or 43C1 (minimally displaced extension into the ankle joint); (2) IM nailing versus direct plating; (3) outcomes including complications, clinical results, and/or function scores; and (4) was a randomized and nonrandomized comparative study. The exclusion criteria were: (1) abstracts, letters, or meeting proceedings; (2) repetitive data; or (3) enrolled patients had pathologically or metabolically induced fractures.

### Search strategy

A computer search of PubMed (1975 to March 2013), Embase (1980 to November 2014), the Cochrane Central Register of Controlled Trials (CENTRAL, November 2014) Chinese Wan-Fang Database (1992 to November 2014), and China National Knowledge Infrastructure (CNKI) (1986 to November 2014) was performed according to the guidelines in the Cochrane Handbook [39]. The following keywords were used: “distal tibia fracture”, “distal* *or* metaphys*”, “fracture fixation”, “fixation, intramedullary fracture”, and “fracture fixation *or* plate *or* plating *or* plates *or* nails *or* nailing.” The search was refined to include clinical RCTs or clinical trials in adult humans. The language of the publication was not restricted. Additionally, we manually searched the reference lists of the included studies for potentially eligible studies. When the same population was reported in several publications, we decided that only the most informative article or the most complete report should be retained to avoid duplication of information.

### Data extraction

Two of the authors independently extracted all available and relevant data from the included studies. A third reviewer resolved any disagreements. The following data were included in the meta-analysis: (1) demographic information, country, study design, interventions, type of fracture, time of the last follow-up, and rate of follow-up; (2) postoperative complications and pain; and (3) functional outcomes. Several corresponding authors were contacted by e-mail to obtain missing information from their publications.

The primary outcomes were infection, delayed union, malunion, removal of instrumentation, secondary procedures, and pain. Infection was classified as deep infection, delayed wound healing, or superficial infection. Delayed union was defined as healing that took longer than 6 months. Malalignment was defined as an axial angulation of more than 5°, an angular rotation of more than 10°, or shortening of more than 1 cm. Secondary outcomes included the functional scores or questionnaires.

### Assessment of study quality

Two authors assessed the risk of bias for each eligible study. The final qualification for each study was determined by consensus among three authors. We evaluated the RCTs according to the Cochrane risk of bias tool [39, 40], which defines seven aspects: (1) randomization; (2) allocation concealment; (3) blinding of participants and implementers; (4) blinding of outcome assessment; (5) incomplete outcome data; (6) selective outcome reporting; and (7) other sources of bias. The risk of bias was qualified as low risk, unclear risk, or high risk. The methodologic qualities of the non-RCT studies (including controlled clinical trials and observational studies) were assessed using the methodologic index for nonrandomized studies (MINORS). MINORS is a valid instrument used to assess the methodologic qualities of nonrandomized surgical studies, including observational studies [41]. We also used the Grading of Recommendations Assessment, Development and Evaluation (GRADE) system to evaluate the quality of evidence by main outcomes in the article.

### Statistical analysis

Statistical analysis was performed using RevMan software (version 5.1; Cochrane Collaboration, Copenhagen, Denmark) for outcome measurements. A value of *p* < 0.05 was considered statistically significant. Heterogeneity was evaluated by visually inspecting the forest plot (analysis) combined with the results of the test for heterogeneity and the *I*^2^ statistic [42]. *I*^2^ > 50 % was considered to be substantial heterogeneity. A fixed-effects model was used in the meta-analysis unless significant heterogeneity existed among the studies. Otherwise, the random-effects model of DerSimonian and Laird [43] was used. Continuous variables were presented as the mean difference (MD), whereas dichotomous variables were presented as the relative risk (RR). Both variables had 95 % confidence intervals (CIs). Sensitivity analysis was performed by deleting a single study at each step to examine the influence of individual data sets on the pooled RRs in the random-effects model. Subgroup analysis was stratified according to the study design. Publication bias was tested using funnel plots whenever possible.

## Results

### Study selection and characteristics

**Fig. 1:**
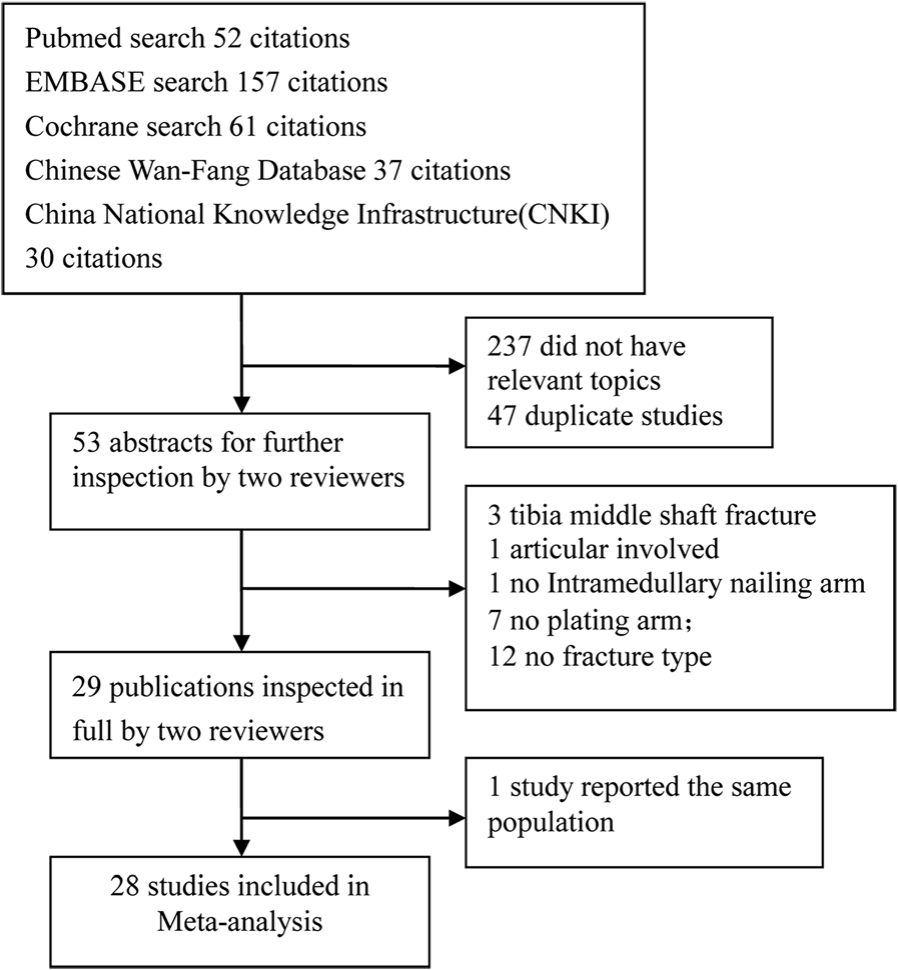
Study selection and inclusion process

The initial search retrieved 337 studies. After examining the titles, abstracts, and full text of the short-listed papers, 29 studies [2, 9–35, 38] were identified as suitable. Two studies used the same population database [34, 35]. We selected outcomes mainly from the later study because it was more informative [35]. Thus, 28 studies were identified at last. The literature selection process is illustrated in Fig. 1. The characteristics and demographic data of each included study are summarized in Table 1.

**Table 1:** Characteristics of included studies

Study	Study design	No. of patients (I *V.* P)	Mean age (year) (I *V.* P)	Female (%)	Fracture types	GAT	Comparisons		Follow-up (month)	ROF (%)
							IM nailing	Plating		
Im et al. 2005 [10]	RCT	64 (34 *V.* 30)	42 *V.* 40	28.1	OTA 43-A1, -A2, -A3, -C1	Closed, or type 1	ACE tibial/cannulated nails	Anatomic plates	24	100
Guo et al. 2010 [33]	RCT	85 (44 *V.* 41)	44.2 *V.* 44.4	35	OTA 43-A1, -A2, -A3	Closed	S2 nailing system	Percutaneous locking compression plates	12	76.6
Vallier et al. 2012 [35]	RCT	104 (56 *V.* 48)	38.1 *V.* 38.5	18.3	OTA 42-A, -B, -C	Closed, type 1, 2, or 3A	Intramedullary nails	Nonlocking plates	12–71	96.2
Mauffrey et al. 2012 [2]	RCT	24 (12 *V.* 12)	50 *V.* 33	33.3	OTA 42-A, -B, -C and OTA43-A	Closed, or type 1	Intramedullary nails	Percutaneous locking plates	12	100
Li 2014 [38]	RCT	82 (40 *V.* 42)	44 *V.* 43	15.3	OTA 42-A, -B, -C	Closed, type 1, 2	Intramedullary nails	Plate	14.8	88.3
Yang et al. 2006 [11]	RP	27 (13 *V.* 14)	54.6 *V.* 48.2	44.4	OTA 43-A	Closed	Shortened intramedullary nails	Nonlocking plates	33 (16–60)	100
Janssen et al. 2007 [13]	RP	24 (12 *V.* 12)	40.8 *V.* 43.3	50	OTA 42-A and -B	Closed, or type 1	Intramedullary nails	Plates	20–112	100
Zhang 2007 [16]	RP	51 (27 *V.* 24)	42.7 *V.* 39.5	39.2	AO A1-3,C1	Closed, type 1, 2	Intramedullary nails	Plates	21.2 (12–27)	100
Vallier et al. 2008 [9]	RP	113 (76 *V.* 37)	38.4 *V.* 39.8	30	OTA 42-A, -B, and -C	Closed, type 1, 2, 3A, or 3B	Intramedullary nails	Nonlocking plates	24 (12–84)	100
Chen et al. 2008 [17]	RP	46 (25 *V.* 21)	31	17.4	AO A and B	Closed	Intramedullary nails	Plates	12–36	100
Huang 2008 [18]	RP	57 (30 *V.* 27)	42.7 *V.* 39.5	36.8	AO A and B	Closed, type 1, 2	Intramedullary nails	Plates	21.8 (10–28)	100
Ni 2010 [19]	RP	57 (32 *V.* 25)	45.8 *V.* 48.0	33.3	AO A1, A2, A3	Closed, type 1, 2, 3A,	Intramedullary nails	Plates	25 (12–33)	96.5
Feng 2011 [32]	RP	50 (22 *V.* 28)	43 V. 45	40	AO A1, A2, A3, C1	Closed, type 1, 2	Intramedullary nails	Locking compression plate	22.8 (12–48)	100
Wu 2011 [20]	RP	43 (25 *V.* 18)	46 *V.* 44	41.9	AO A1, B1, B2, C1	Closed, type 1, 2	Intramedullary nails	Locking compression plate	16.2 (10–22)	100
Huang 2012 [21]	RP	52 (26 *V.* 26)	41.7 *V.* 42.0	44.2	AO 42A1-3, 43A1, 43A2	Closed, type 1, 2	Intramedullary nails	Locking plate	12	100
Jin 2012 [22]	RP	170 (72 *V.* 98)	47.5 *V.* 44.5	42.9	AO 41A, 42B, 43A-C	NA	Intramedullary nails	Locking compression plate	8–14	100
Li 2012 [14]	RP	46 (23 *V.* 23)	37 *V.* 39	21.7	AO 43A1-3	Closed, type 1, 2	Locked nailing	Locking compression plate	24.7 *V*. 25.8	100
Ren 2012 [23]	RP	58 (28 *V.* 30)	31.9 *V.* 32.4	34.5	AO A, B, and C	Closed, type 1, 2	Intramedullary nails	Plates	6–36	100
Seyhan et al. 2012 [12]	RP	61 (25 *V.* 36)	40.3 *V.* 39.7	44.4	OTA 42-A, -B, and -C	Closed, type 1, 2, or 3A	Expert, Synthes, and Trigen (Smith and Nephew) nails	Percutaneous locking plates	21.24 (12–60)	100
Tan 2012 [24]	RP	96 (48 *V.* 48)	43.7 *V.* 44.6	43.8	AO A1-3, B1	Closed and Open	Intramedullary nails	Plate	12–24	100
Yang 2012 [25]	RP	32 (17 *V.* 15)	39	40.6	AO 42A B	Closed, type 1, 2	Intramedullary nails	Locking plate	15.1 (14–20)	100
Ke 20113 [26]	RP	62 (32 *V.* 30)	45.8 *V.*47.3	37.1	AO A1, A2, A3	Closed, type 1, 2	Intramedullary nails	plate	12	100
Wang 2013 [27]	RP	98 (47 *V.* 51)	42.7 *V.* 40.1	NA	OTA 43A	NA	Intramedullary nails	Plate	12	96.1
Yao 2013 [28]	RP	126 (65 *V.* 61)	49.2 *V.* 48.0	38.9	OTA 42 A-C,43A	Closed, type 1, 2, or 3A	Intramedullary nails	Locking compression plates	23.7 (12–53)	100
Zhu 2013 [29]	RP	74 (37 *V.* 37)	43.7 *V.* 44.1	45.9	43A 1-3	Closed and Open	Intramedullary nails	Plates	6 (3–12)	100
Dong 2014 [30]	RP	46 (22 *V.* 24)	35.6 *V.* 37.3	28.3	AO A, B, and C	Closed	Intramedullary nails	compression plates	8–36	100
Guo 2014 [31]	RP	60 (30 *V.* 30)	45.2 *V.* 44.5	41.7	AO A1-A3	Closed	Intramedullary nails	Locking compression plates	12	100
Yavuz 2014 [15]	RP	55 (21 *V.* 34)	38 *V.* 44	41.8	OTA 42 A-C	Closed type 1, 2	Intramedullary nails	Plates	27.6 (12–82)	100

*GAT* Gustilo and Anderson Type, *I* V. *P* IM nailing versus plating, *ROF* rate of follow-up, *V.* versus, *RCT* randomized controlled trial, *RP* retrospective

A total of 1863 patients with distal tibia fractures were included in the meta-analysis. Five RCTs and 23 retrospective studies were performed from 2005 to 2014. The total number of patients in each study ranged from 24 to 170. The percentage of female patients in the study populations ranged from 15.3 to 50.0 %. The studies followed patients from 6 to 112 months. The rate of patient follow-ups ranged from 76.6 to 100 %.

### Study quality

**Fig. 2:**
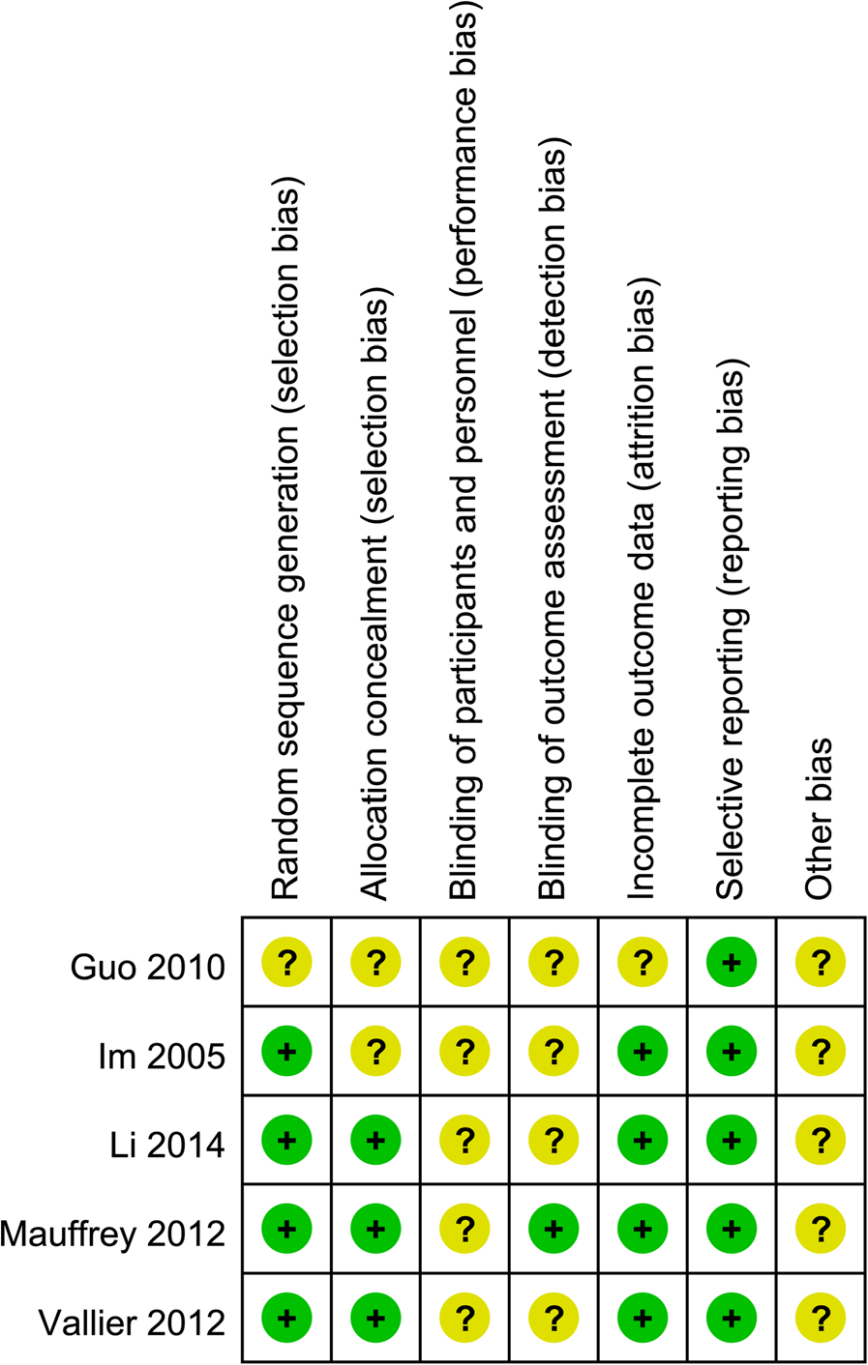
Risk of bias: summary for randomized controlled trials. *plus sign* low risk, *question mark* unclear risk

The methodologic quality of the included RCTs is assessed in Fig. 2. Randomized sequences were generated in four RCTs [2, 10, 35, 38] by drawing envelopes [10] or computer randomization [2, 35, 38]. One study [33] did not clearly describe the random sequence generation. Three trials used opaque envelopes [35, 38] or central allocation [2] for concealment. The blinding of participants was not mentioned in any of the studies. The outcome assessors were blinded in the study by Mauffrey et al. [2]. Three studies had complete outcome data [2, 10, 38], whereas Vallier et al. [35] used an “intention to treat” analysis. The study by Guo et al. [33] had missing data, balanced in numbers across intervention groups, but did not report the reasons for the loss of participant follow-up. None of the included studies used selective reporting. The other sources of bias remained unclear. The MINORS quality scores of the retrospective studies are presented in Table 2. The mean score was 16.74 (range 16–18), which corresponded to a 69.8 % score. The most obvious limitations of the previous studies are the lack of blinding, prospective collection of data, and prospective calculation of the study size. The GRADE analysis showed the moderate and low quality in the main outcomes (Tables 3 and 4). The most common reasons for the decreased level of evidence were suspected publication bias because of inadequate included original studies. Heterogeneity also reduced the evidence grade of the grip strength, the range of motion (ROM), and the radiological results.

**Table 2:** MINORS appraisal scores for the included retrospective studies

Study	Methodologic items^a^												Total
	1	2	3	4	5	6	7	8	9	10	11	12	
Yang et al. 2006 [11]	2	0	0	2	0	2	2	0	2	2	2	2	16
Janssen et al. 2007 [13]	2	1	0	2	0	2	2	0	2	2	2	2	17
Zhang 2007 [16]	2	1	0	2	0	2	2	0	2	2	2	1	16
Vallier et al. 2008 [9]	2	2	0	2	0	2	2	0	2	2	2	2	18
Chen et al. 2008 [17]	2	1	0	2	0	2	2	0	2	2	2	2	17
Huang 2008 [18]	2	1	0	2	0	2	2	0	2	2	2	2	17
Ni 2010 [19]	2	1	0	2	0	2	2	0	2	2	2	2	17
Feng 2011 [32]	2	1	0	2	0	2	2	0	2	2	2	2	17
Wu 2011 [20]	2	1	0	2	0	2	2	0	2	2	2	1	16
Huang 2012 [21]	2	1	0	2	0	2	2	0	2	2	2	2	17
Jin 2012 [22]	2	0	0	2	0	2	2	0	2	2	2	2	16
Li 2012 [14]	2	1	0	2	0	2	2	0	2	2	2	2	17
Ren 2012 [23]	2	1	0	2	0	2	2	0	2	2	2	1	16
Seyhan et al. 2012 [12]	2	0	0	2	0	2	2	0	2	2	2	2	16
Tan 2012 [24]	2	1	0	2	0	2	2	0	2	2	2	2	17
Yang 2012 [25]	2	1	0	2	0	2	2	0	2	2	2	2	17
Ke 20113 [26]	2	1	0	2	0	2	2	0	2	2	2	2	17
Wang 2013 [27]	2	0	0	2	0	2	2	0	2	2	2	2	16
Yao 2013 [28]	2	1	0	2	0	2	2	0	2	2	2	2	17
Zhu 2013 [29]	2	1	0	2	0	2	2	0	2	2	2	2	17
Dong 2014 [30]	2	1	0	2	0	2	2	0	2	2	2	2	17
Guo 2014 [31]	2	1	0	2	0	2	2	0	2	2	2	2	17
Yavuz 2014 [15]	2	1	0	2	0	2	2	0	2	2	2	2	17

^a^Methodologic items: (1) a clearly stated aim; (2) inclusion of consecutive patients; (3) prospective collection of data; (4) endpoints appropriate to the aim of the study; (5) unbiased assessment of the study endpoint; (6) follow-up period appropriate to the aim of the study; (7) loss to follow up, which is less than 5 %; (8) prospective calculation of the study size; (9) an adequate control group; (10) contemporary groups; (11) baseline equivalence of groups; and (12) adequate statistical analyses. The items are scored as “0” (not reported), “1” (reported but inadequate), or “2” (reported and adequate). The global ideal score for comparative studies is 24 [41]

**Table 3:** The GRADE evidence quality for complications

Complications	Number of studies	Study design	Risk Ratio [95 % CI]	*P*	*P* for heterogeneity	Quality
Deep infection	4 [2,10,35,38]	RCT	0.79 [0.27, 2.29]	0.67	0.72	Moderate^a^
	4 [9,12,28,29]	Retrospective	0.44 [0.14, 1.41]	0.17	0.43	Moderate^b^
Delayed wound healing and superficial infection	4 [2,10,33,38]	RCT	0.41 [0.11, 1.61]	0.20	0.13	Moderate^a^
	14 [12,14-19,21,24-28,31,32]	Retrospective	0.34 [0.21, 0.57]	<0.0001	0.61	Moderate^b^
Delayed union	4 [2,10,35,38]	RCT	1.46 [0.70, 3.03]	0.31	0.37	Moderate^a^
	8 [9,12,13,17,19,28-30]	Retrospective	0.99 [0.62, 1.59]	0.98	0.72	Low
Removal of metal work	3 [2,33,35]	RCT	0.89 [0.62, 1.27]	0.51	0.39	Moderate^a^
	6 [9,12,13,17,23,27]	Retrospective	0.89 [0.36, 2.17]	0.79	0.08	Low
Secondary procedures	4 [2,33,35,38]	RCT	0.92 [0.62, 1.37]	0.69	0.13	Moderate^a^
	6 [9,12,13,17,23,27]	Retrospective	0.78 [0.33, 1.80]	0.55	0.02	Low
Malunion	4 [2,10,35,38]	RCT	1.52 [0.81, 2.85]	0.20	0.64	Moderate^a^
	14 [9,11-14,16,18,20,22,24,26,28,29,31,32]	Retrospective	4.79 [2.86, 8.01]	<0.00001	1.00	Moderate^c^
Knee pain	2 [35,38]	RCT	5.39 [0.13, 229.08]	0.38	0.009	Low^a,d^
	6 [11,13-15,28,29]	Retrospective	4.01 [1.71, 9.40]	0.001	0.14	Moderate^c^

^a^Total number of events is less than 300

^b^RR < 0.5

^c^RR > 2

^d^ *I* ^2^ > 50 %

**Table 4:** The GRADE evidence quality for functional outcomes

Functional outcomes	Number of studies	Study design	Mean difference [95 % CI]	*P*	*P* for heterogeneity	Quality
Olerud and Molander Ankle Score	4 [14,16,21,25]	Retrospective	0.01 [−0.02, 0.03]	0.56	0.09	Low
American Orthopaedic Foot and Ankle Surgery score	2 [27,28]	Retrospective	4.10 [0.03, 8.17]	0.05	0.10	Low
Radiologic union	3 [10,33,38]	RCT	−0.53 [−2.39, 1.34]	0.58	<0.00001	Low^a,b^
	8 [14,16,20,22,24,25,29,32]	Retrospective	−0.98 [−3.61, 1.66]	0.47	<0.00001	Low

^a^Total number of events is less than 300

^b^ *I* ^2^ > 50 %

### Primary outcomes

Our meta-analysis did not suggest a statistically significant difference between the two treatments in terms of the rate of deep infection, delayed union, removal of instrumentation, or secondary procedures in the RCT and retrospective study subgroups (Table 3).

**Fig. 3:**
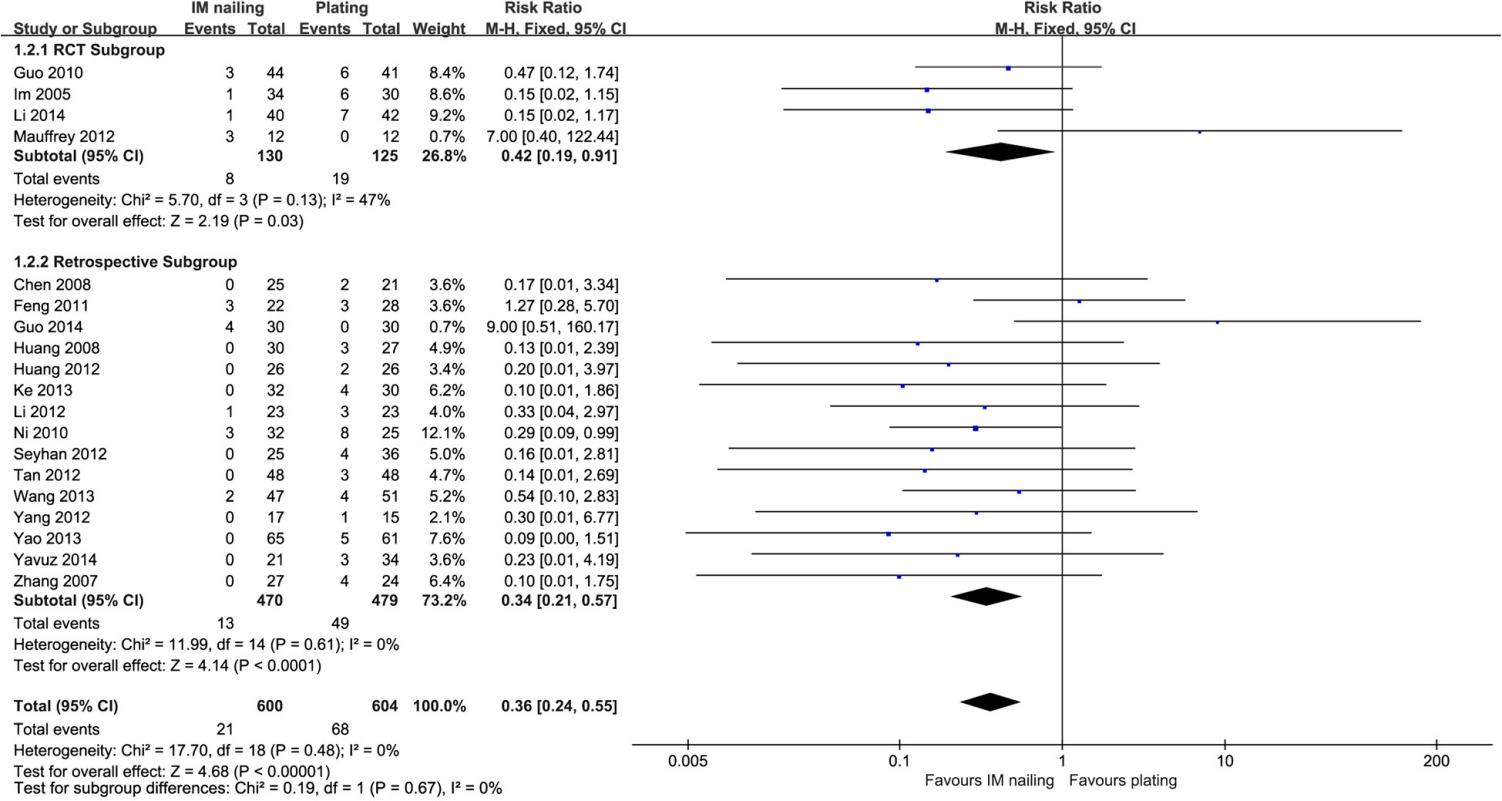
Delayed wound healing and superficial infection: IM nailing versus plating

Nineteen studies, with 1204 fractures, reported delayed wound healing and superficial infection [2, 10, 12, 14–19, 21, 24–28, 31–33, 38]. Plating was associated with significantly higher rate of delayed wound healing and superficial infection both in RCT subgroups and the retrospective subgroups with 95 % CIs of 0.19–0.91 (*p* = 0.03) and 0.21–0.57 (*p* < 0.0001), respectively (Fig. 3).

**Fig. 4:**
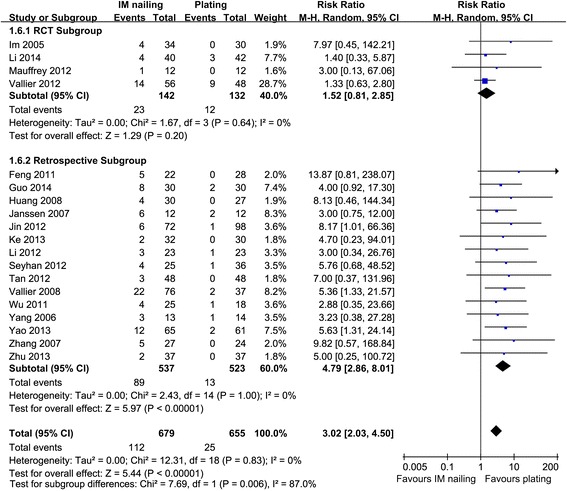
Malunion: IM nailing versus plating

Nineteen studies, with 1334 fractures, reported malunion [2, 9–14, 16, 18, 20, 22, 24, 26, 28, 29, 31, 32, 35, 38]. IM nailing was associated with significantly more malunions in the retrospective subgroup and across all studies, with 95 % CIs of 2.86–8.01 (*p* < 0.00001) and 2.03–4.50 (*p* = 0.006), respectively. The malunion rate did not differ significantly in the RCT subgroup, with a 95 % CI of 0.81–2.85 (*p* = 0.20) (Fig. 4).

**Fig. 5:**
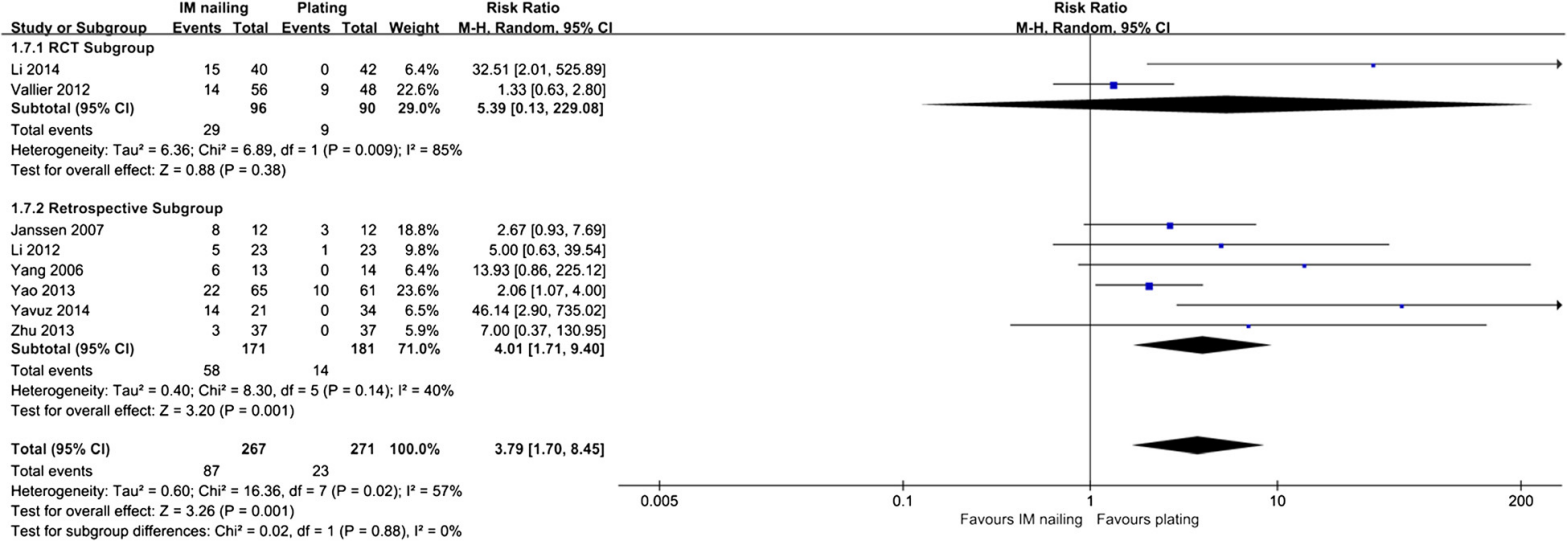
Knee pain: IM nailing versus plating

Two RCTs [35, 38] and six retrospective studies [11, 13–15, 28, 29] reported knee pain. IM nailing was associated with a significantly higher incidence of knee pain in the retrospective subgroup and across all studies, with 95 % CIs of 1.71–9.40 (*p* = 0.001) and 1.70–8.45 (*p* = 0.001), respectively (Fig. 5).

### Secondary outcomes

Several functional scores were used in the included studies, including the Olerud and Molander Ankle Score (OMAS) [14, 16, 21, 25], American Orthopaedic Foot and Ankle Surgery scores (AOFAS) [27, 28, 33], EuroQol EQ-5D [2], Disability Rating Index (DRI) [2, 44], Musculoskeletal Function Assessment (MFA) [34, 35], and Foot Function Index (FFI) [34, 35]. According to our results, these differences were not statistically significant regarding OMAS and AOFAS (Table 4). Mauffrey et al. [2] used the DRI, OMAS, and EuroQol EQ-5D as outcome measures. They reported that at 6 months, the IM nails achieved a difference of 13 points in the DRI compared with the plates in favor of the use of IM nails, but the difference was not statistically significant (*p* = 0.498). Yang et al. [11] found that similar results were seen with OMAS (*p* = 0.644).

### Publication bias

**Fig. 6:**
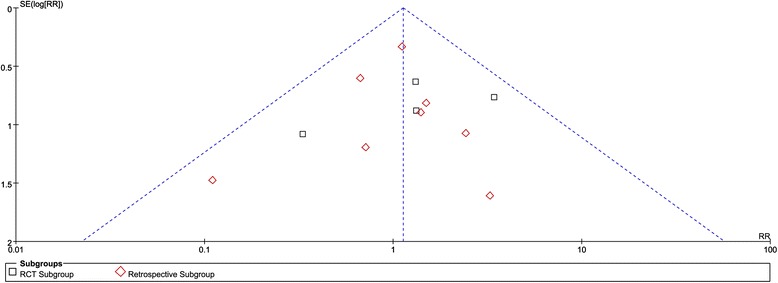
Funnel plot for delayed union

For the meta-analysis of delayed union, there was no evidence of significant publication bias by inspection of the funnel plot (Fig. 6).

## Discussion

To compare IM nailing with plating for distal tibia fractures without articular involvement and to determine the dominant strategy, we performed a meta-analysis of RCTs and observational studies. There were no significant differences in the incidence of deep infection, delayed union, removal of instrumentation, or secondary procedures. Compared with plating, IM nailing was associated with significantly more malunions, a higher incidence of knee pain in the retrospective subgroup and across all of the studies but was not significant in the RCT subgroup, and a lower rate of delayed wound healing and superficial infection both in the RCT and retrospective subgroup. Also, functional scores did not support a significant difference between the two implants.

We updated the previous meta-analysis published recently, including five RCTs, of which two articles reported the same trial and no subgroup analysis was made. The informative one was selected as two studies used the same population database [34, 35], and what is more, new RCT and observational studies were added in the present meta-analysis. We also used GRADE approach, which makes clinical guidelines easier for users to assess the judgments behind recommendations to conclude the quality of evidence of the main outcomes. A meta-analysis of RCTs is generally considered to provide the highest level of evidence for clinical interventions. However, RCTs are rare in orthopedics. Therefore, observational studies were included in our study for the best available evidence [45]. Subgroup analysis was included in the study design because the included observational studies had an inherent risk of bias. Thus, the present study not only provides answers to clinical questions and can serve as the basis for practice guidelines, it thoroughly describes the state of the literature on a specific question and can help direct future research [45].

The results of the RCT and retrospective subgroups were not consistent in regard to malunion or knee pain. The inconsistency may be due to several factors. First, the sample size was insufficient. In the RCT subgroup, only four studies in the meta-analysis reported malunion and two reported knee pain. Second, the retrospective studies often overestimated the treatment effects because of selection bias. In this meta-analysis, the results for the RCT subgroup, the retrospective subgroup, and the total studies were presented to provide a more detailed description of the evidence from comparative studies. Combining the RCT and retrospective groups amplified the sample size, thereby increasing the statistical power. Conversely, the results were representative of all of the comparative studies and thoroughly describe the current state of evidence. Doing so, however, affects the strength of the conclusion. The selection biases that exist in retrospective studies were thus combined in one data pool. Therefore, the results should be interpreted with caution.

The biomechanics of plating distal tibia fractures are superior to those of IM nailing because a plate construct is nearly twice as stiff as an IM nail under an axial load [3, 46]. A previous study, however, suggested that plating distal tibia fractures was often associated with a high risk of soft tissue complications, such as deep and superficial infections and delayed wound healing [3]. Hence, we included these risks as primary outcomes, and the desired product was synthesized in the meta-analysis. No significant differences were found in terms of deep infections associated with the two implants, either in the pairwise subgroup or overall analysis. An open fracture is at significant risk of developing a deep infection, but not with either nailing or plating [12, 35]. The risk of delayed wound healing or superficial infection showed a significant difference (*p* < 0.00001) favoring IM nailing in subgroups and across all studies.

Another problem associated with plating with direct or indirect reduction was implant irritation, which usually prompted removal of the instrumentation and the need for a secondary procedure [1]. Implant irritation may be related to the high profile of anatomically contoured plates [1]. The rate of removal was previously reported to be as high as 52 % [47]. A similar trend favored IM nailing in our meta-analysis, although there were no significant differences across all groups or within the two subgroups. The rates of instrumentation removal were 16 and 20.1 % for the nailing and plating groups, respectively (*p* = 0.71). Their rates for performance of secondary procedures were 24.1 and 30 %, respectively (*p* = 0.40).

Delayed union and malunion have been the most debated complications. Achieving and maintaining good reduction with IM nailing is notably difficult [12, 13] because of the anatomic characteristics of distal tibia fractures. This disadvantage of IM nailing is believed to contribute to delayed union and malunion [3]. A previous retrospective study similarly reported high rates of malunion and nonunion [9]. However, a number of implants and surgical advances have been developed to improve IM nailing durability and to aid fracture reduction [1, 14], including blocking screws and multiple-plane locking screws. We found no significant differences in the delayed union rates. However, the results of our subgroup analysis of the rates of malunion were internally inconsistent within the subgroup. The retrospective subgroup analysis showed a significant difference in the rate of malunion between IM nailing and plating. The weakness of the study design could have caused the observed bias, although it remains unclear if that was the case.

Previous studies have commonly reported rates of knee pain ranging from 19.0 to 73.2 % for IM nailing [47–49]. In our meta-analysis, the rate of knee pain was 32.6 % for nailing versus 8.5 % for plating. The results from the pairwise subgroup analysis were similarly inconsistent. The only two RCTs that showed the rate of knee pain did not have significant differences, although the retrospective subgroup and total group analysis significantly favored plating. The retrospective studies often overestimated the treatment effects of inherent limitations. In addition, another RCT used the pain score as an outcome and found no significant differences. Several studies suggested that the incidence of knee pain after nailing may be decreased by applying certain techniques, such as protecting the patellar tendon, avoiding damage to the anterior intermeniscal ligament, and minimizing nail prominence [35, 48].

Reports of the functional scores and questionnaires from the two implants varied within the literature. No significant difference was observed between two methods regarding OMAS and AOFAS. Only one study [10] reported a significant range of movement in favor of IM nailing. Mauffrey et al. [2] suggested that the IM nailing group recovered quickly (within 6 months after surgery). Future studies should use validated instruments for functional primary outcome measurements. Finally, the various scoring systems and questionnaires should be effectively evaluated. We also failed to find any difference in terms of radiologic union.

The high heterogeneity was caused by the different study designs. This issue was solved using subgroup analysis, achieved by dividing the studies into RCTs or non-RCTs. The residual heterogeneity could have been induced by poor study design. Other potential sources of heterogeneity are the different plates used and the fibular fracture fixation. Locking and nonlocking plates were combined into one meta-analysis group and then compared with IM nailing. A recent literature review showed similar complication rates among locking and nonlocking plates [50]. No obvious heterogeneity was found between these plates. Thus, we did not analyze locking and nonlocking plates in the subgroups. Another debated factor was fibular fracture fixation. A recent RCT reported that fixation of fibular fractures had no effect on nonunion or malunion of tibia fractures [51]. Subgroup analysis was not possible because the included trials did not provide data in separate groups for these interventions and fibular fracture fixation. Therefore, fibular fracture fixation was not analyzed in the present study.

The data from the present meta-analysis showed that IM nailing may be associated with malunion and knee pain but with low rate risk of delayed wound healing or superficial infection. Some advice on IM nailing may be useful: first, the fracture configuration should be considered. The distal fragment should have enough bone volume to receive and hold at least two screws. Second, surgeons should master the techniques of using a straight guidewire, blocking screws, and multiple-plane locking screws. These instruments can be used effectively for avoiding malalignment and malunion. Third, a suitable IM nailing length and careful treatment of soft tissue may be useful for minimizing the incidence of knee pain. Fourth, IM nailing may be suitable for particular populations, such as patients for whom there is concern about wound healing (e.g., older patients with thin skin, diabetic patients with skin problems) [1, 3]. Patients in our meta-analysis were mainly young, healthy adults at the time of their injury.

Plating can achieve almost anatomic reduction and stable fixation as it can include more distal and smaller fragments. These advantages make plating suitable for almost all distal tibia fractures when soft tissue injury is not a consideration [1]. Although the results for deep infection problems did not differ significantly in our study, plates should be used cautiously in patients with potential soft tissue problems. Future studies should focus on more special injury patterns.

The present analysis clearly had limitations. First, only five RCTs with 420 fractures could provide level I evidence, so the total number of high-level RCTs in the analysis was relatively small. The modest sample sizes also decreased the power of the pooled estimates. Therefore, large and rigorous RCTs are required. Second, both RCTs and non-RCTs were included in this study. The retrospective studies often overestimated the treatment effects. Thus, results should be interpreted with caution.

## Conclusion and implications for future research

Evidence suggests that both IM nailing and plating are appropriate treatments as IM nailing shows lower rate of delayed wound healing and superficial infection while plating avoids malunion and knee pain. Large, rigorous RCTs are required for determining the optimal treatment because of the modest sample sizes and the heterogeneity among the studies’ designs. The choice of treatments should be based on the surgeon’s expertise, the clinical circumstances, and especially the patient’s injury pattern.

## Abbreviations

IM: Intramedullary
RCTs: Randomized controlled trials
CNKI: China National Knowledge Infrastructure
MINORS: Methodologic index for nonrandomized studies
GRADE: Grading of recommendations assessment, development and evaluation
CIs: Confidence intervals
OMAS: Olerud and Molander Ankle Score
AOFAS: American Orthopaedic Foot and Ankle Surgery scores
DRI: Disability Rating Index
MFA: Musculoskeletal Function Assessment
FFI: Foot Function Index
